# Sinusoidal Obstruction Syndrome Following Myeloablative Therapy and Tranexamic Acid Treatment for Hemorrhage in Two Patients with Neuroblastoma

**DOI:** 10.3390/children7110198

**Published:** 2020-10-28

**Authors:** Felix Zirngibl, Carina Flemmig, Peter Lang, Annette Künkele, Angelika Eggert, Johannes H. Schulte, Hedwig E. Deubzer

**Affiliations:** 1 Department of Pediatric Hematology and Oncology, Charité—Universitätsmedizin Berlin, 13353 Berlin, Germany; felix.zirngibl@charite.de (F.Z.); carina.flemmig@charite.de (C.F.); annette.kuenkele@charite.de (A.K.); angelika.eggert@charite.de (A.E.); johannes.schulte@charite.de (J.H.S.); 2 Berliner Institut für Gesundheitsforschung (BIH), 10178 Berlin, Germany; 3 Parexel International GmbH, 14050 Berlin, Germany; 4 Department of Pediatrics, Children’s University Hospital, University of Tübingen, 72076 Tübingen, Germany; peter.lang@med.uni-tuebingen.de; 5 German Cancer Consortium (DKTK), Partner Site Berlin, 10117 Berlin, Germany; 6 German Cancer Research Center Heidelberg (DKFZ), 69120 Heidelberg, Germany; 7 Experimental and Clinical Research Center (ECRC), Charité—Universitätsmedizin Berlin and the Max-Delbrueck-Center for Molecular Medicine (MDC) in the Helmholtz Association, 13125 Berlin, Germany

**Keywords:** pediatric hematopoietic stem cell transplantation, embryonal tumor, transplant-related complication, fibrinolysis inhibitor, thrombotic event

## Abstract

Adverse thromboembolic events following administration of the anti-fibrinolytic agent tranexamic acid (TA), used to prevent/treat excessive blood loss, are rare. We present the clinical course of two young patients (22 and 56 months) receiving busulfan/melphalan (Bu/Mel) high-dose chemotherapy with autologous hematopoietic stem cell transplantation (HSCT) to treat high-risk neuroblastoma, who developed hepatic sinusoidal obstruction syndrome (SOS) within 48 h after systemic TA treatment for a hemodynamically relevant hemorrhage. Defibrotide treatment resolved hepatic SOS, but the short time between TA administration and SOS onset suggests a causal association.

## 1. Introduction

Hepatic sinusoidal obstruction syndrome (SOS), previously known as hepatic veno-occlusive disease, is a non-hematological complication of the chemo- or radiotherapy-based conditioning regimens used in the allogeneic and, less frequently, autologous hematopoietic stem cell transplantation (HSCT) setting [1]. The primary event causing this potentially life-threatening syndrome affects the sinusoidal endothelial cells surrounding the central vein in the hepatic acini [1,2]. The complex cascades triggering injury and sinusoidal endothelial cell activation in the liver include the glutathione-based detoxification of chemotherapeutics and their metabolites, cytokine release by tissues damaged through the conditioning regimen and hemostatic system activation [2]. Occlusion of the venular lumen, post-sinusoidal hypertension eventually causing retrograde portal venous flow and hepatocellular necrosis lead, untreated in severe cases, to liver failure, hepatorenal syndrome and multisystem organ failure, and ultimately to death in ≥80% of cases [1]. First described in 1979 [3,4], SOS is diagnosed in HSCT recipients by applying the European Society for Blood and Marrow Transplantation (EBMT) diagnostic criteria for adults [1] or Baltimore criteria [5]/(modified) Seattle criteria [6], which include the day of onset after HSCT, hyperbilirubinemia ≥2 mg/dL (~34 µmol/L), tender hepatomegaly and/or unexplained weight gain greater than 5% over baseline by fluid retention in the third space. The EBMT diagnostic criteria for children additionally include weight gain and rising bilirubin over 3 consecutive days, respectively, as well as unexplained consumptive and transfusion-refractory thrombocytopenia [7]. The different diagnostic criteria are shown side by side in Table 1.

## 2. Results

**Figure 1:**
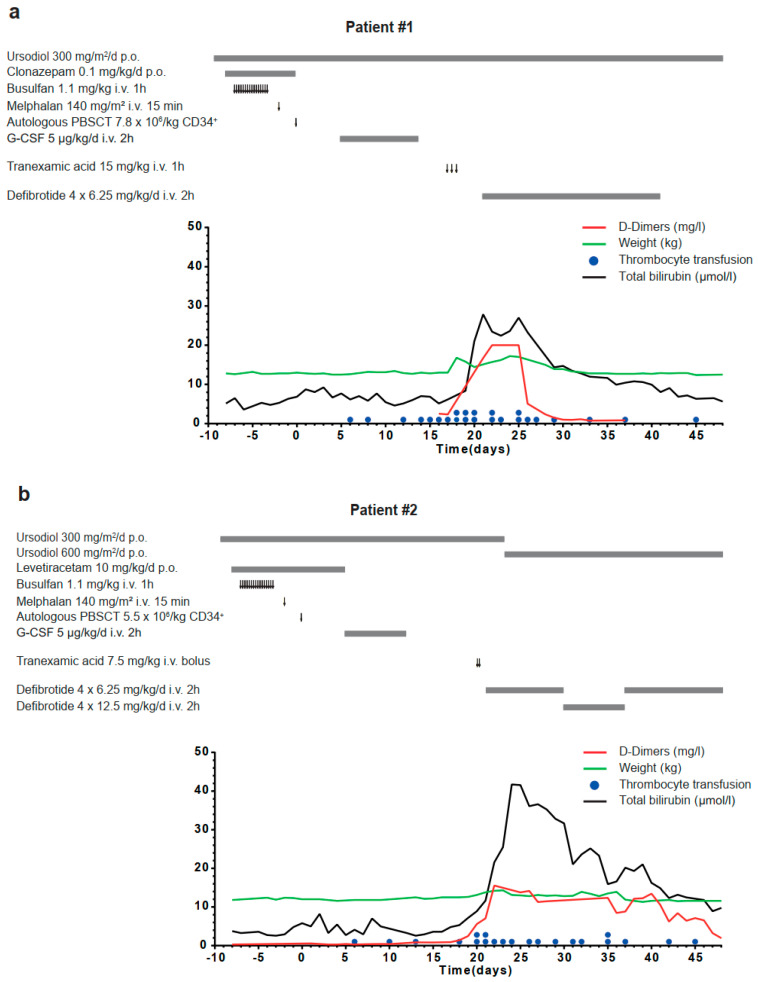
Schematic diagram illustrating medical interventions, clinical signs and laboratory parameters of a 4-8/12-year-old INRG stage M neuroblastoma patient (**a**) and a 1-10/12-year-old INRG stage M neuroblastoma patient (**b**) receiving myeloablative chemotherapy and autologous stem cell rescue. Arrow, therapy application; bold grey line, treatment interval; red line, D-dimer values (mg/L); black line, total bilirubin level (mmol/L); green line, body weight (kg); blue dots, thrombocyte transfusions. CD: cluster of differentiation; G-CSF: granulocyte colony-stimulating factor; INRG: International Neuroblastoma Risk Group; PBSCT: peripheral blood stem cell transplantation.

The 56-month-old child was treated according to the NB2004 High-Risk Trial protocol for International Neuroblastoma Risk Group (INRG) stage M neuroblastoma without MYCN amplification, with six cycles of antineoplastic polychemotherapy followed by complete resection of the primary tumor arising from the left adrenal gland. Complete remission was verified by magnetic resonance imaging (MRI), iodine-123-metaiodobenzylguanidine scintigraphy and bone marrow aspiration before consolidation treatment with high-dose chemotherapy (consisting of Bu/Mel) and autologous HSCT. On day 14, macrohematuria mediated by a BK virus cystitis was diagnosed. On day 17, bleeding caused a drop in hemoglobin level from 10 to 7 g/dL and, ultimately, grade IV vesical tamponade developed despite intensive intravenous hydration with >5000 L/qm^2^ body surface. To halt extensive blood loss from multiple mucosal lesions in the urinary bladder that were too diffuse to be treated by vascular obliteration, 15 mg tranexamic acid (TA)/kg body weight was systemically administered in three single doses at 12-h intervals (Figure 1a). According to Corbacioglu S et al. [7], the EBMT criteria for diagnosing SOS in children were fulfilled ~48 h later, namely tender hepatomegaly, weight gain >5% above baseline due to ascites and rising bilirubin on 3 consecutive days (Figure 1a). In addition, thrombocytopenia with rapid platelet consumption and an increase in D-dimers above 20.00 mg/L were observed (Figure 1a). Bilirubin levels peaked at only 1.63 mg/dl (~27.72 µM) (Figure 1a). Defibrotide treatment was immediately initiated and administered for a total of 21 days at 6.25 mg/kg body weight in 6-h intervals (Figure 1a). According to EBMT criteria for grading SOS severity in children [7], the patient developed a very severe SOS due to the rapid bilirubin kinetics and prolonged refractory thrombocytopenia. The patient fully recovered by day 30.

The 22-month-old child was treated according to the NB Registry 2016 for INRG stage M neuroblastoma with MYCN amplification. After induction treatment and complete resection of the primary tumor, consolidation treatment followed, with high-dose chemotherapy (Bu/Mel) and autologous HSCT. A total of 15 mg TA/kg body weight was systemically administered in two boluses within 2 h on day 20 in response to a hemodynamically relevant hemorrhage from the insertion channel after central venous catheter exchange. Within 24 h, the patient developed right upper quadrant pain, weight gain >5% above baseline, consumptive thrombocytopenia and showed rising serum bilirubin on 3 consecutive days, reaching >2 mg/dL after 96 h. Thus, the EBMT diagnostic criteria for children were fulfilled on day 21, and defibrotide treatment was initiated (Figure 1b). According to EBMT criteria for grading SOS severity in children [7], the patient developed a very severe SOS due to the rapid bilirubin kinetics and greater than eight-fold increased transaminases combined with the need for replacement of coagulation factors. The patient recovered only after intensified and prolonged continuation of defibrotide by day 48 (Figure 1b).

The children were treated at a single institution that conducts approximately 50 allogeneic and autologous transplantations per year. To date, 15 children with neuroblastoma have received an autologous HSCT after Bu/Mel conditioning at our department since June 2016. The two patients described in this case report developed SOS in close temporal context to TA administration, and one patient developed a grade 4 SOS without prior treatment with TA. The remaining 12 patients did not develop SOS and none of them received TA during the period around autologous HSCT.

## 3. Conclusions

TA competitively blocks the lysine-binding sites of plasminogen, plasmin and tissue plasminogen activator [8] to reversibly impede fibrinolysis and blood clot degradation. TA is mainly used to prevent or treat excessive blood loss after liver transplantation [9] and multiple trauma [10]. Several risk factors for SOS development are present in both patients, namely, both were diagnosed with neuroblastoma and both received myeloablative conditioning containing busulfan before autologous HSCT. We suggest that the temporal relationship between the use of TA and SOS onset, rather, indicates a causal and not coincidental connection, although additional evidence is needed to prove TA treatment as a risk factor for SOS. Animal models of SOS provide a suitable platform for further studies [11,12]. Thus, the systemic application of TA to prevent bleeding complications early after myeloablative therapy should be carefully considered due to increasing evidence for a potentially additive role in SOS development [13], especially in patients with pre-existing high-risk features for hepatic SOS. Tandem myeloablative consolidation therapy has been shown to improve survival probability in patients with high-risk neuroblastoma [14], emphasizing the importance of minimizing transplant-related morbidity.

## Figures and Tables

**Table 1:** Different diagnostic criteria for SOS in adults and children.

Baltimore Criteria for Adults, 1987 [5]	Modified Seattle Criteria for Adults, 1984 [6]	EMBT Criteria for Children, 2018 [7]
Bilirubin serum level >2 mg/dL and at least 2 of following within 20 days of transplant: Hepatomegaly Ascites Weight gain >5% from baseline	Two of following occurring within 21 days after HSCT: Hepatomegaly or right upper quadrant pain Bilirubin serum level >2 mg/dL Unexplained weight gain >2% from baseline	No limitation for time of SOS onset The presence of two or more of the following: Unexplained consumptive and transfusion-refractory Thrombocytopenia Otherwise unexplained weight gain on 3 consecutive days despite the use of diuretics or a weight gain >5% above baseline value Hepatomegaly Ascites Rising bilirubin from a baseline value on 3 consecutive days or ≥2 mg/dL within 72 h

EBMT: European Society for Blood and Marrow Transplantation; HSCT: hematopoietic stem cell transplantation; SOS: sinusoidal obstruction syndrome.
